# Peripheral thermal responses in normal and cold-sensitive individuals to sublingual Glyceryl Trinitrate (GTN)

**DOI:** 10.1186/2046-7648-4-S1-A34

**Published:** 2015-09-14

**Authors:** Katrina Hope, Clare M Eglin, Frank Golden, Michael J Tipton

**Affiliations:** 1 School of Physiology & Pharmacology, Clinical Research and Imaging Centre (CRICBristol), University of Bristol, 60 St Michael´s Hill, Bristol, BS2 8DX, UK; 2 Extreme Environments Laboratory, Department of Sport and Exercise Science, University of Portsmouth, Portsmouth, UK

## Introduction

Non-freezing cold injury (NFCI) is caused by prolonged exposure of the extremities to cold. The long-term sequelae of NFCI, include cold-sensitivity and pain[1]. The cold sensitivity is characterised by a reduction in basal skin blood flow and augmented vasoconstriction during cold exposure. We tested the hypothesis that sublingual GTN would increase blood flow in the peripheral microcirculation during and after a mild cold challenge in individuals who had not been diagnosed with NFCI, but were cold-sensitive.

## Methods

In air at 30 °C, seven control and six cold-sensitive participants undertook 12 min of gentle exercise prior to immersing their right foot (protected by a thin plastic bag) into 15 °C water for 2 min, followed by 10 min of spontaneous rewarming. Two minutes prior to immersion, participants were given either 400 µg GTN or placebo sublingually in a single-blinded, counter-balanced order. Toe pad skin temperature (T_sk_) and blood flow (SkBF) were measured using infrared thermography and laser Doppler flowmetry respectively.

## Results

In the placebo condition, T_sk _was significantly lower in Cold-sensitive participants compared to controls throughout the test (P < 0.001) as was SkBF (P < 0.05).

**Figure 1:**
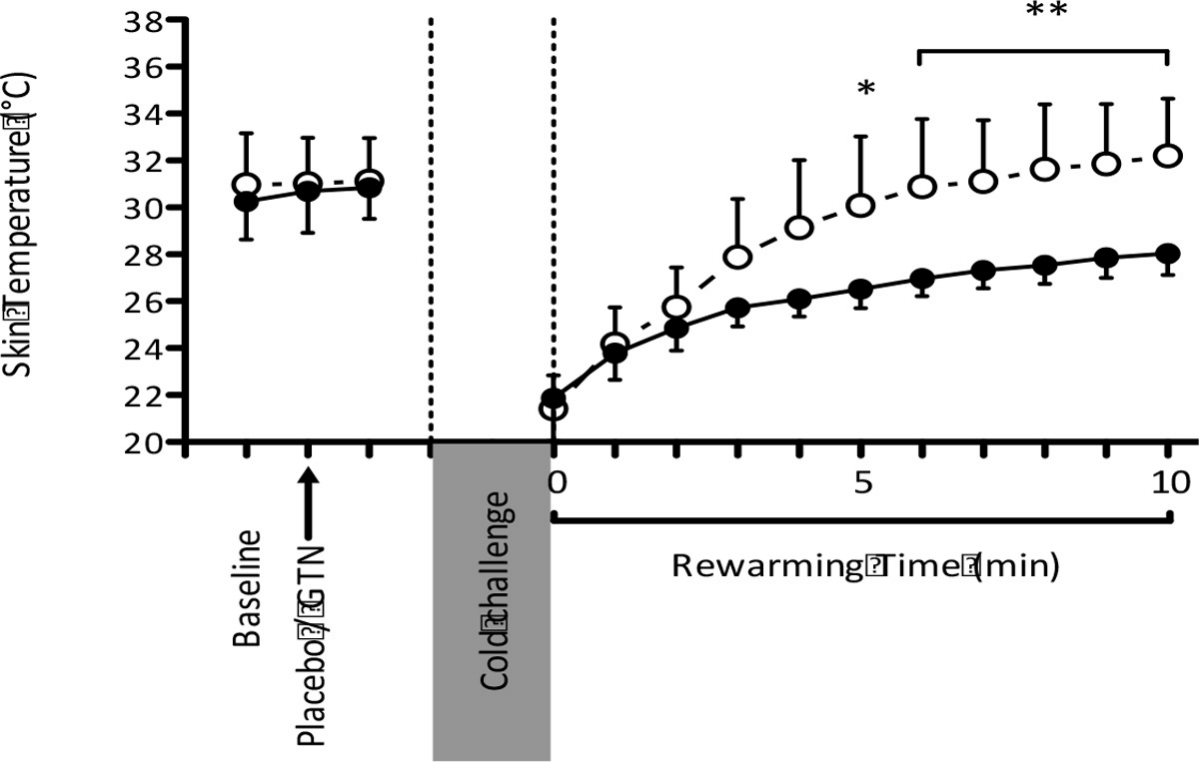
**Mean (SD) T_sk _of coldest toe before and after foot immersion in 15 °C water in Cold-sensitive group following placebo (l) or GTN (¡)** * P < 0.05, ** P < 0.001

GTN increased the rate of rewarming (°C.min^-1^) and absolute T_sk _of the coldest toe after the cold challenge in Cold-sensitive (placebo: 0.62(0.14) °C.min^-1^, 28.03(0.92) °C; GTN: 1.08(0.29) °C.min^-1^, 32.20(2.43) °C; p < 0.001) but not control individuals (Figure 1). GTN also increased the blood flow in the great toe during rewarming in some cold-sensitive individuals.

## Discussion

We accept our hypothesis that impairment in the vasodilatory response seen in individuals with cold-sensitivity can be overcome by the use of GTN, an endothelial-independent nitric oxide donor, and thereby improve the rewarming of cooled peripheral tissues.

## Conclusion

Individuals with cold-sensitivity show increased vasoconstrictory tone, both at rest and during warming after a cold stimulus, compared to controls. The use of GTN to overcome this implies an abnormal endothelium and nitric oxide pathway in this condition.
